# Umbilical Cord Mesenchymal Stem Cells Ameliorate Inflammation-Related Tumorigenesis via Modulating Macrophages

**DOI:** 10.1155/2022/1617229

**Published:** 2022-06-01

**Authors:** Yanxia Fu, Jun Li, Mengdi Li, Junfeng Xu, Zheng Rong, Fangli Ren, Yinyin Wang, Jianqiu Sheng, Zhijie Chang

**Affiliations:** ^1^Department of Biochemistry and Molecular Biology, Capital Medical University, Beijing 100069, China; ^2^State Key Laboratory of Membrane Biology, School of Medicine, Institute of Precision Medicine, Tsinghua University, Beijing 100084, China; ^3^TsCell Biotech Inc., Beijing 100084, China; ^4^Senior Department of Gastroenterology, The First Medical Center of Chinese PLA General Hospital, Beijing 100853, China; ^5^Department of Gynaecology and Obstetrics, Jishuitan Hospital, Beijing 100096, China; ^6^Department of Gastroenterology, The Seventh Medical Center of PLA General Hospital, Beijing 100700, China

## Abstract

Mesenchymal stem cells (MSCs) have been documented to be effective for the therapy of inflammation-related diseases but raised concerns on possible tumorigenic effects. Since most of the tumors are induced or promoted by chronic inflammation, one could expect that MSCs might be beneficial for the cancer therapy because of their potent roles on inhibiting inflammation. This study is aimed at performing a safety evaluation and evaluating the role of human umbilical cord mesenchymal stem cells (HUC-MSCs) on tumorigenesis. We found that HUC-MSCs cultured within 20 generations had no significant changes in proliferation, cell cycle, cellular senescence, apoptosis, and expression of mesenchymal stem cell markers. HUC-MSCs were unable to form any tumor in immunodeficiency or normal mice with or without inflammatory stimulation. Intriguingly, we observed that HUC-MSCs inhibited tumorigenesis in B16-derived or AOM/DSS-induced colon cancer models. We reasoned that the effect of HUC-MSCs on tumorigenesis might be through regulating the inflammatory response. Indeed, HUC-MSCs dramatically ameliorated the disease symptoms and pathological changes of DSS-induced colitis mice. We deciphered the mechanism that HUC-MSCs inhibited tumorigenesis through reducing the proportion of macrophages, which were decreased in the mice suffered from AOM/DSS-induced colon cancer. Correspondingly, the expression levels of TNF-*α* and IL-6, which were secreted by macrophages, were significantly decreased in the plasma of colon cancer and colitis mice after injection of HUC-MSCs. This study revealed the role of inhibiting macrophages and shed light on the therapeutic application of HUC-MSCs in inflammation-induced tumorigenesis.

## 1. Introduction

Colitis-associated colon cancer is one of the most common fatal malignancies worldwide and the second leading cause of cancer-related death in developed countries. Environmental, genetic, infectious, inflammation, and immune factors have been identified for the occurrence and development of colitis-associated colon cancer [1]. It has been widely accepted that the chronic inflammation is one of the most important risk factors for tumorigenesis. Chronic inflammation could function as a driving force for tumorigenesis by inducing gene mutations, enhancing proliferation, and resisting apoptosis [2]. Inflammation is caused by a persistently heightened immune response following injury or exposure to foreign pathogens. Under inflammation conditions, immune cells including T, B, NK, and macrophages were dysregulated. In particular, macrophages in the gastrointestinal tract play pivotal roles in the maintenance of mucosal homeostasis, although the unbalance of Treg, Th1, Th2, and Th17 cells is also functional. All these cells secreted proinflammatory factors, which promote tumor growth. Macrophages contribute to cancer initiation by producing proinflammatory mediators such as IL-6 and TNF-*α*, proteases, reactive oxygen species (ROS), and nitrogen species that may create a mutagenic microenvironment [3]. However, M2 macrophages secrete IL-10 to exert anti-inflammatory effects [4].

To date, the treatment on colon cancer remains far from satisfactory. Novel therapeutic strategies are needed to improve the efficiency and reduce the side effects. Mesenchymal stem cell- (MSC-) based therapies have provided convincing evidence for treating a variety of inflammatory and autoimmune diseases with the potential anti-inflammatory and immunomodulatory effects [5]. Many studies have demonstrated that MSCs are effective to alleviate rhinitis [6], arthritis [7], colitis [8], systemic lupus erythematosus nephritis [9], and graft-versus-host disease [10]. Currently, MSCs have been used in more than 100 clinical trials worldwide to treat a wide variety of diseases [11]. A possible mechanism is that MSCs might be able to migrate to the inflammation site where they regulate the function of immune cells. MSCs restrain activation and proliferation of T cells, B cells, and NK cells; repress dendritic cell maturation; promote the generation of regulatory T cells (Treg cells); induce macrophage differentiation from proinflammatory M1 phenotype to anti-inflammatory M2 phenotype; and reduce inflammation by secreting of IL-10 [12–18]. MSCs suppress the secretion of inflammatory factor TNF-*α* and IL-6, reducing inflammation. Intriguingly, MSCs have been reported to suppress hepatoma through the Wnt signaling pathway [19], inhibit lung cancer cell proliferation [20], and suppress glioma growth through inhibition of angiogenesis [21]. On the other hand, bone marrow-derived MSCs were reported to promote tumor growth in breast cancer and prostate cancer by increasing the expression of phosphate forming factor [22]. Nevertheless, these controversial observations raised a question that MSCs might function on tumorigenesis under different conditions.

Due to the fact that chronic inflammation is an important factor for the progression of colon cancer, we hypothesized that MSCs may suppress colitis-associated colon cancer through repressing inflammation. In this study, we report that MSCs from human umbilical cord (HUC-MSCs) are effective for the therapy of tumors. We showed that HUC-MSCs reduce macrophages, as well as T cells, and reduce the inflammatory cytokines, including TNF-*α* and IL-6. The observation provided a clue that HUC-MSCs might inhibit tumorigenesis by regulation of inflammatory responses via macrophages.

## 2. Materials and Methods

### 2.1. Animals

Healthy C57BL/6J, ICR, and BALB/c nude mice were all obtained from Vitalriver Company (Beijing, China). The mice were maintained in a pathogen-free room and fed with an autoclaved pellet diet and water ad libitum. All mice were housed in isolated ventilated cages (six mice per cage) with barrier facility at Tsinghua University, Beijing China. The mice were maintained on a 12/12-hour light/dark cycle, under 22-26°C with sterile pellet food and water ad libitum. The laboratory animal facility has been accredited by AAALAC (Association for Assessment and Accreditation of Laboratory Animal Care International). All animal protocols used in this study were approved by the IACUC (Institutional Animal Care and Use Committee) of Tsinghua University, Beijing China. All efforts were made to minimize the number of animals used and to reduce their suffering.

### 2.2. Cell Cultures

HUC-MSCs were cultured with high-glucose Dulbecco-modified Eagle medium (DMEM) (Gibco, Grand Island, N Y) supplemented with 2 mM L-glutamine, 5% fetal bovine serum (FBS, Gibco), 100 U/ml penicillin, and 100 *μ*g/ml streptomycin (Gibco) and cytokines (EGF, bFGF, PDGF, and IGF). Cultures were maintained at 37°C in a humidified atmosphere with 5% CO_2_. Adherent spindle-shaped cells were cultured to 80% confluence then trypsinized using 0.25% trypsin (Gibco) and passaged in the medium described above.

### 2.3. Immunophenotypic Analysis of HUC-MSCs

To analyze the expression of classical MSC markers, cells were inspected by flow cytometry analysis using antihuman antibodies against CD29, CD31, CD34, CD44, CD45, CD90, CD105, HLA-DR (human leukocyte antigens-DR), CD181 (CXCR1), CD182 (CXCR2), CD184 (CXCR4), CD195 (CCR5), and CD197 (CCR7) (purchased from eBioscience or BioLegend, USA). For flow cytometry analyses, MSCs were stained with antibodies for 20 min on ice in the dark, washed twice, and resuspended in PBS supplemented with 1% FCS. For control, unstained cells and IgG isotype antibody were used. FACS Arial II (Becton Dickinson, USA) instrument and FlowJo software were used for acquiring and analysis of the samples.

### 2.4. Multilineage Differentiation Analysis *In Vitro*

Differentiation analysis was carried out to demonstrate the ability of HUC-MSCs differentiated into adipocytes and chondrocytes *in vitro*. HUC-MSCs at passage 5 were cultured under conditional media (STEMCELL) to induce the differentiation of chondrocytes or adipocytes according to the manufacturers' protocol. Oil Red O and Alcian Blue were used to stain adipocytes and chondrocytes, respectively.

### 2.5. Cell Cycle and Apoptosis Analysis

For cell cycle analysis, 1 × 10^6^ of different passages of HUC-MSCs were fixed with ice-cold methanol. The cells were treated with 100 *μ*g/ml DNase-free RNase A and stained with 10 *μ*g/ml propidium iodide for 30 min on ice, prior to analysis by a flow cytometer. For apoptosis analysis of different passages of HUC-MSCs, the cells were stained with Annexin V and PI (BioLegend, USA) before analysis with flow cytometry.

### 2.6. Colitis Induction

Experimental colitis in mice was induced by 3.5% (wt/vol) DSS (MP Biochemicals, USA) in sterile drinking water for 6 days [23]. One day before DSS treatment, mice were randomly divided into two groups and injected intravenously with either 2 × 10^6^ cells in 200 *μ*l PBS per mice (*n* = 7) or with 200 *μ*l PBS alone (*n* = 7) at day 1, day 3, and day 5. Healthy mice fed with a normal diet and sterile water were used as controls. Stool consistency, fecal bleeding, and weight loss were used to evaluate the severity of colitis [23]. The full colon was removed, and its length was measured at the end of the experiment. Then, colon tissues were fixed in 4% formaldehyde overnight and transferred to 75% ethanol. After embedding in paraffin, longitudinal sections of the entire colon were prepared for histological studies.

### 2.7. Intestinal Permeability Evaluation *In Vivo*

The intestinal permeability was assessed by FITC-labeled dextran as described [18]. In brief, mice (*n* = 7, per group) were gavaged with FITC-dextran (Mw 4000, Sigma-Aldrich, USA) at a concentration 60 mg/100 g body weight 7 days after DSS treatment. Blood was collected 4 h after gavage of FITC-dextran. The amount of FITC-dextran in serum was measured with a fluorescence spectrophotometer (emission and excitation wavelengths: 485 and 530 nm).

### 2.8. Analysis of Macrophages in the Colon

To isolate lymphocytes, the colon was taken out and removed off the mesentery, Peyer's patches, fat, and content. Then, the colon was moved into the medium (RPMI 1640, 10% FBS, 1% P/S, 5 mM EDTA, 20 mM HEPES) to allow cells separated by shaking in a 37°C incubator at 190 rpm for 30 min. The remaining tissue after washing off the epithelial cells was minced and digested with 10 U/ml collagenase CLISPA (Worthington Biochemical, USA) and 0.1 mg/ml DNase I at 37°C for 40 min. Subsequently, heavy-density cells were purified in 40% Percoll (Sigma, USA) by centrifugation for 10 min at 800 × g.

### 2.9. Cytokine Measurement

Mice were anesthetized by pentobarbital sodium, and blood samples were collected. TNF-*α*, IL-6, IFN-*γ*, IL-4, IL-17, and TGF-*β*1 levels in serum were detected using ELISA kits (eBioscience or BioLegend, USA) according to the manufacturer's instructions.

### 2.10. Tumorigenesis Assay *In Vivo*

The mouse B16 cell line (mouse melanoma cells) cultured in DMEM and 10% fetal calf serum and H22 cells (mouse H22 hepatocarcinoma cells) isolated from mouse ascites were used to evaluate the ability of tumorigenesis. Balb/C nude mice aged at 4 weeks (*n* = 7, per group) were injected subcutaneously with 1 × 10^6^ of B16 cells or HUC-MSCs into the left and right flanks. And 5 × 10^6^ H22 cells or HUC-MSCs were injected into ICR mice (*n* = 6, per group), respectively. Two weeks after injection, mice were sacrificed and tumors were separated for measurement.

### 2.11. Statistical Analysis

All data are expressed as means ± SD. Comparisons between two groups were performed using the Student *t* test, whereas differences among >2 groups were performed using one-way ANOVA with GraphPad Prism version 5.0 statistical software. Differences were considered significant if *p* < 0.05.

## 3. Results

### 3.1. Characterization and Safety Evaluation of HUC-MSCs Isolated from Human Umbilical Cord

**Figure 1:**
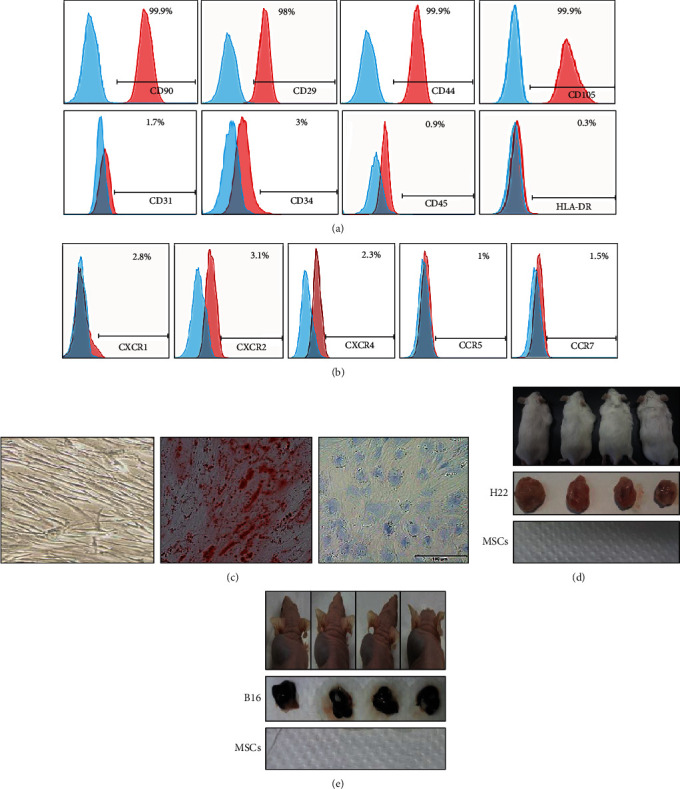
Characterization and the safety evaluation of HUC-MSCs isolated from Wharton jelly of the umbilical cord. (a) Identification of the expression of MSC markers by flow cytometry. CD29, CD44, CD90, and CD105 were highly expressed in HUC-MSCs, while CD31, CD34, CD45, and HLA-DR were lowly expressed. (b) HUC-MSCs express detectable chemokine receptors. (c) Multilineage differentiation potential of HUC-MSCs. HUC-MSC control (left); Oil Red O staining for adipogenic differentiation (middle); Alcian Blue staining for osteogenic differentiation. To make sure of the safety of HUC-MSC injection, tumor-bearing experiment was used in immunodeficiency and normal immunity mice. Error bars: mean ± SD. ^∗^*p* < 0.05; ^∗∗^*p* < 0.01; ^∗∗∗^*p* < 0.005. (d) The same amount of HUC-MSCs and H22 cells (5 × 10^6^ cells) was, respectively, injected into the left and right side of ICR mice to compare tumor formation ability of HUC-MSCs and H22 cells (*n* = 6). (e) The same amount of HUC-MSCs and B16 cells (1 × 10^6^ cells) was, respectively, injected into the right and left side of nude mice to compare tumorigenesis ability *in vivo* (*n* = 7).

To seek for an effective therapy for inflammation-related colon cancer, the stemness of HUC-MSCs was characterized in the 5th generation. The results showed that more than 98% of the cells expressed CD90, CD29, CD44, and CD105, markers of stem cells, whereas less than 3% of the cells expressed CD31, CD34, and CD45, markers of endothelial or hematopoietic (Figure 1(a)). Intriguingly, we observed that the percentage of HLA-DR, a marker of immune rejection response, was only 0.3% in the cells (Figure 1(a), last panel). These results suggest that the isolated HUC-MSCs retained the features of mesenchymal stem cells but were latent to stimulate immune responses. As MSCs have been reported to home to inflammation or injury sites as these cells express several chemokine receptors^4^, we determined to examine whether HUC-MSCs express chemokine receptors. FACS analyses showed that CXCR1 and CXCR2 were moderately, but CXCR4, CCR5, and CCR7 were slightly expressed by HUC-MSCs (Figure 1(b)). These results suggest that HUC-MSCs might have a moderate homing ability.

To examine the differentiation ability of HUC-MSCs, we treated the isolated HUC-MSCs with conditional medium. The results showed that HUC-MSCs were able to differentiate into adipocytes and chondrocytes *in vitro* (Figure 1(c)). Importantly, in order to examine whether HUC-MSCs have tumorigenesis potential, we inoculated HUC-MSCs on the mice. B16 and H22 cells were used as controls. The results showed that HUC-MSCs failed to form any tumor while both B16 and H22 formed large tumors (Figures 1(d) and 1(e)). These results indicate that HUC-MSCs retain partial characteristics of mesenchymal stem cells and are unable to form tumors.

### 3.2. The Number of Passages of HUC-MSCs without Affecting the Characteristics of MSCs

**Figure 2:**
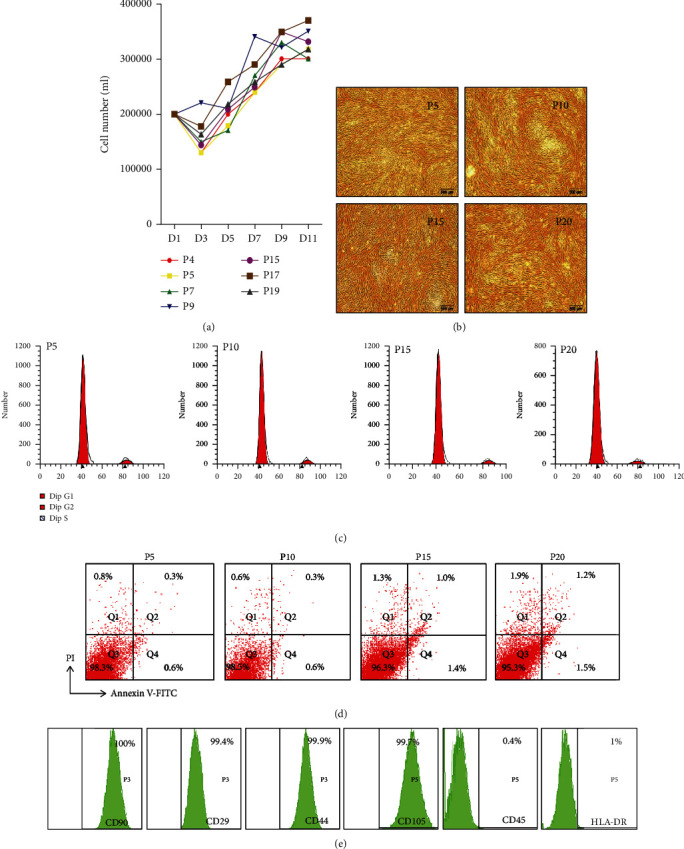
The passages of HUC-MSCs without affecting the characteristics of stem cells. (a) Proliferation curve of HUC-MSCs. There were little changes in the proliferation rate of HUC-MSCs from passage 4 to passage 19. (b) Cellular senescence assay of different passages of HUC-MSCs. (c) Cell cycle of different passages of HUC-MSCs. (d) Apoptosis analysis of different passages of HUC-MSCs. (e) Identification of MSC markers for the 20th-generation HUC-MSCs by flow cytometry.

A cell proliferation assay indicated that HUC-MSCs showed similar abilities of proliferation during early 20 generations (Figure 2(a)). Furthermore, we observed that HUC-MSCs retained the same features of cellular senescence (Figure 2(b)), cell cycle (Figure 2(c)), and apoptosis (Figure 2(d)) among passages 5, 10, 15, and 20. The results of flow cytometry showed that mesenchymal stem cell markers remained of no significant change when HUC-MSCs were passed to the 20th generation (Figure 2(e)). These results suggest that the HUC-MSCs that we isolated could be cultured within 20 generations.

### 3.3. The Effect of HUC-MSCs on Colitis-Associated Tumorigenesis in Mice

**Figure 3:**
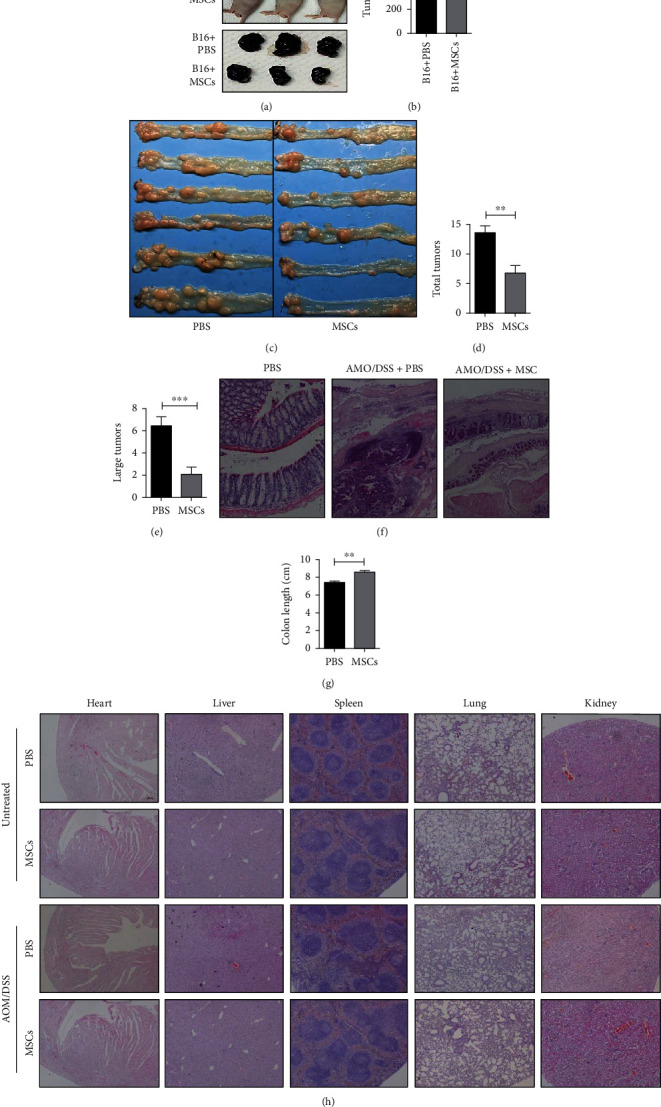
HUC-MSCs effectively inhibit tumorigenesis. (a, b) The same amount of B16 cells (1 × 10^6^ cells) was injected into the left side of PBS and HUC-MSC group nude mice; then, 1 × 10^6^ HUC-MSCs were intravenously injected into the MSC group twice a week, and the same amount of PBS was injected into the PBS group to observe the occurrence of tumors (*n* = 6). (c) HUC-MSC injection reduces colonic tumor burden in C57BL/6J mice induced by AOM/DSS (*n* = 5). (d, e) Tumor numbers in the PBS and HUC-MSC group after treatment with AOM/DSS. (f) Hematoxylin-eosin (HE) staining of colons. Intravenous injection of HUC-MSCs promotes histological improvement. (g) Colon length in the PBS and HUC-MSC group after treatment with AOM/DSS. (h) Pathologic section of the colon, heart, liver, spleen, lung, and kidney in the PBS and HUC-MSC group treated with AOM/DSS. HUC-MSCs were intravenously injected once a week. No tumors were found in the heart, liver, spleen, lung, and kidney injected with or without HUC-MSCs.

To address whether HUC-MSCs have any potential to promote tumorigenesis, we inoculated B16 cells in the nude mice with or without injection of HUC-MSCs. The results showed that B16 cells formed significant tumors, but after injection of HUC-MSCs, tumor growth is partially inhibited (Figures 3(a) and 3(b)). This observation promoted us to study whether HUC-MSCs have a long-term effect on tumor formation. For this end, we generated AOM/DSS-induced colon cancer mice and applied HUC-MSC treatment. The results demonstrated that AOM/DSS induced significant colon cancers (Figure 3(c), left panels); however, the number of tumors in the colon was significantly decreased in the mice intravenously injected with HUC-MSCs in comparison with the PBS group (Figure 3(c)). A quantitative presentation showed that the total tumor number (Figure 3(d)) and large tumor number (Figure 3(e)) were significantly decreased when the mice were injected with MSCs. Histological analyses demonstrated that injection of HUC-MSCs restored the intestinal structure into normal tissues while AOM/DSS treatment altered the intestine into tumor tissues (Figure 3(f)). In addition, the colon length in HUC-MSC-treated mice was also significantly longer than that of PBS-treated mice (Figure 3(g)). These results suggest that HUC-MSCs exert an antitumor effect in AOM/DSS-induced colon cancer. Furthermore, no tumor was observed in the heart, liver, spleen, lung, and kidney (Figure 3(h)), and no significant changes were found in the heart, liver, spleen, lung, and kidney index (Fig. S1) in mice injected with HUC-MSCs. Taken together, we concluded that injection of HUC-MSCs inhibits tumor growth in xenograft tumor and regresses tumorigenesis induced by AOM/DSS.

### 3.4. Intravenous Injection of HUC-MSCs Protects Mice against DSS-Induced Colitis

**Figure 4:**
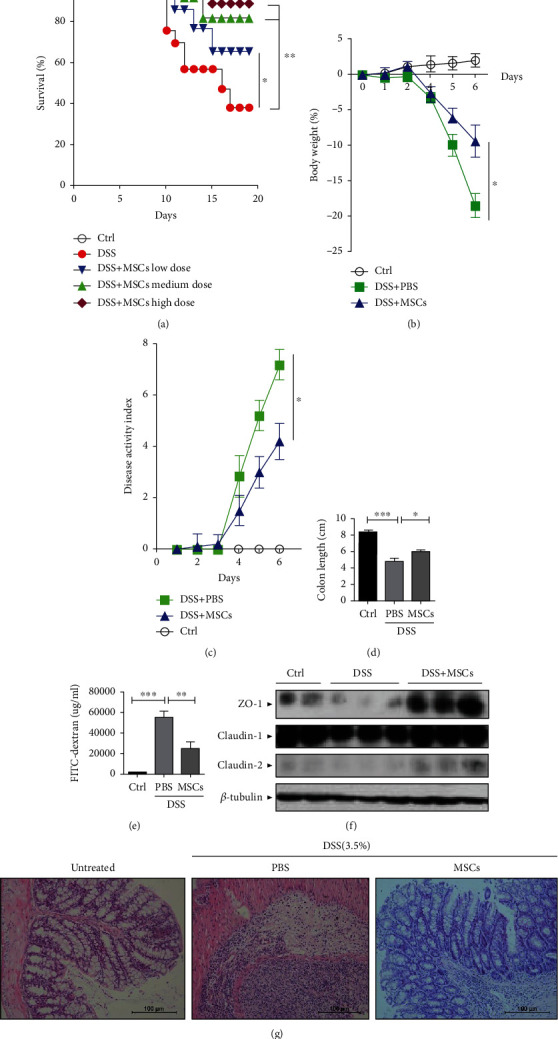
HUC-MSC treatment protects against DSS-induced acute colitis. Colitis was induced by oral administration of 3.5% (wt/vol) DSS in sterile drinking water for 6 days. Mice were injected intravenously with 2 × 10^6^ HUC-MSCs in 200 *μ*l phosphate-buffered saline (PBS) per mouse in the cell injection group or with 200 *μ*l PBS per mouse in the DSS-treated group at day 1, day 3, and day 5. ^∗^*p* < 0.05; ^∗∗^*p* < 0.01; ^∗∗∗^*p* < 0.005. Error bars: mean ± SD. (a) Survival rate of mice treated with 5% (wt/vol) DSS and different doses of HUC-MSCs. The dose of cells injected into mice was 1 × 10^5^, 2 × 10^5^, and 5 × 10^5^ per mice. (b) Weight changes of mice with or without 3.5% (wt/vol) DSS treatment. (c) Disease activity index (DAI) scores after DSS administration. Clinical evolution was monitored by weight change, stool consistency, and presence of fecal blood. (d) Statistics of colon lengths in normal, DSS-, and HUC-MSC-treated groups. (e) Evaluation of colonic permeability with FITC-dextran in serum of colitis mice. (f) Evaluation of colonic permeability with western blot. (g) Hematoxylin-eosin (HE) staining of colons. Intravenous injection of HUC-MSCs promotes histological improvement. Colon histology demonstrated intravenously administered HUC-MSCs reduced the extent of the inflamed area, crypt damage, and infiltration of inflammatory cells caused by DSS.

We reasoned that the effect of HUC-MSCs on the AOM/DSS-induced colon tumor was due to their role on the inflammation repression. To test this hypothesis, we applied a DSS-induced colitis model. We challenged C57BL/6J mice with DSS and injected HUC-MSCs. The results showed that 76.9% of mice died with 20 days after DSS treatment (Figure 4(a), red dots); however, injections of different dosages of HUC-MSCs *via* tail veins significantly improved the survival rate of mice challenged by DSS in a dose-dependent manner (Figure 4(a), blue, green, and brown dots). The highest dosage of HUC-MSCs obtained the best therapeutic effect (Figure 4(a), brown dots). We then examined body weight and disease activity index (DAI). The results showed that the body weight of mice treated with DSS was significantly elevated by HUC-MSCs compared to that of PBS-treated mice (Figure 4(b)). Correspondingly, injection of HUC-MSCs decreased the disease activity index (Figure 4(c)). The colon length was shortened after DSS treatment but was recovered by injection of HUC-MSCs (Figure 4(d)). The colonic permeability, evaluated by the permeated amounts of the FITC-dextran, was dramatically increased after DSS treatment but the HUC-MSCs reduced the permeability significantly (Figure 4(e)). Consistently, HUC-MSCs significantly recovered the expression of ZO-1, claudin-1, and claudin-2, which were decreased by DSS treatment (Figure 4(f)). Histological analyses demonstrated that DSS treatment disrupted the colon crypts and caused the infiltration of immune cells (Figure 4(g), panels 2 to 1). However, injection of HUC-MSCs dramatically recovered the crypt structure and reduced immunocyte infiltration (Figure 4(g), panels 3 to 2). All the results suggest that HUC-MSCs ameliorated the inflammation of colitis. To address whether HUC-MSCs were able to home to the damaged sites caused by DSS, we used fluorescence-labeled HUC-MSCs. A flow analysis showed that HUC-MSCs were able to home to the colon but also to the spleen, although the ratio in the colon was higher than that in the spleen (Fig. S2). These results suggest that HUC-MSCs might function both at local and whole regulation. Nevertheless, our results suggest that HUC-MSCs have a potent ability to repress inflammation.

### 3.5. HUC-MSCs Regulate Inflammatory Cytokine Production in Colitis and Colitis-Related Colon Cancer

**Figure 5:**
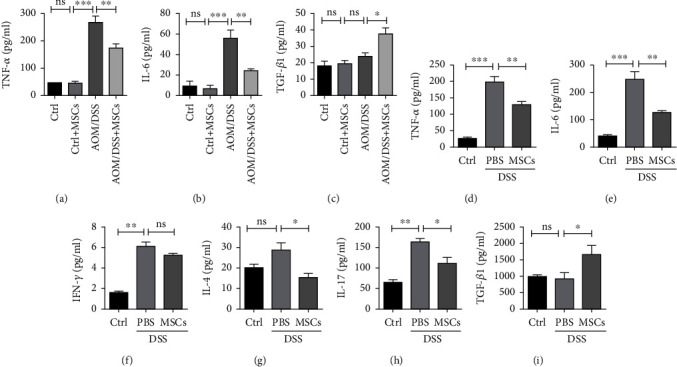
HUC-MSCs regulate inflammatory cytokine production in colitis and colitis-related colon cancer. (a–c) HUC-MSC treatment reduced TNF-*α* and IL-6 while increased TGF-*β*1 in the serum of AOM/DSS-treated mice. (d, e) HUC-MSC treatment reduced proinflammatory cytokines produced by macrophages in serum caused by DSS. (f, g) HUC-MSC treatment reduced IFN-*γ* and IL-4 in serum of colitis mice. (h, i) HUC-MSC treatment reduced IL-17 while increased TGF-*β*1 in serum caused by DSS. Error bars: mean ± SD. ^∗^*p* < 0.05; ^∗∗^*p* < 0.01; ^∗∗∗^*p* < 0.005.

To address how HUC-MSCs regulate inflammation responses, we tested the levels of critical inflammatory cytokines in AOM/DSS-induced tumor mice with HUC-MSC therapy. TNF-*α* and IL-6, secreted by macrophages, were significantly reduced (Figures 5(a) and 5(b)), while TGF-*β*1 was significantly increased by HUC-MSCs after AOM/DSS treatment (Figure 5(c)). These results suggest that HUC-MSCs might regulate immune cells including macrophages to secrete cytokines. We then examine the cytokines produced in DSS-induced colitis mice. The results showed that TNF-*α* and IL-6 were significantly reduced in the serum of DSS-induced mice after HUC-MSC treatment (Figures 5(d) and 5(e)). In addition, we found that DSS-induced secretion of IFN-*γ* (Figure 5(f)) by Th1 cells, IL-4 (Figure 5(g)) by Th2 cells, and IL-17 (Figure 5(h)) by Th17 cells was significantly reduced by the injection of HUC-MSCs. On the other hand, TGF-*β*1, an anti-inflammatory cytokine, secreted by Treg cells, was significantly increased by HUC-MSCs (Figure 5(i)). These results suggest that HUC-MSCs significantly suppress the secretion of proinflammatory cytokines and increase the anti-inflammatory cytokine during colitis and colitis-related colon cancer.

### 3.6. HUC-MSCs Regulate the Immune Cell Populations

**Figure 6:**
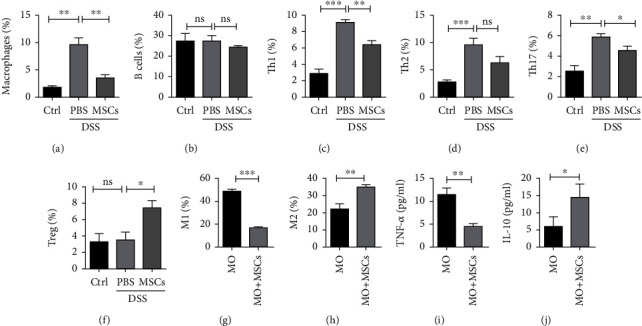
HUC-MSCs regulate immune cell balance. (a) Transplant of HUC-MSCs downregulated activated macrophage in the colon mucosa. With the treatment with DSS, the activated macrophages in the colon mucosa were significantly increased, whereas therapy with HUC-MSCs, the percentage of activated macrophages was decreased greatly. (b) HUC-MSCs have no significant effect on the proportion of B cells in DSS-induced colitis mice. (c, d) Transplant of HUC-MSCs decreased Th1 and Th2 in DSS-induced colitis mice. (e, f) Transplant of HUC-MSCs decreased Th17 while increased Treg in DSS-induced colitis mice. (g–j) The influence of HUC-MSCs on differentiation and secretion of macrophages. Error bars: mean ± SD. ^∗^*p* < 0.05; ^∗∗^*p* < 0.01; ^∗∗∗^*p* < 0.005.

To examine the alteration of immune cells, mice were sacrificed at day 7 after DSS treatment. FACS analyses indicated that HUC-MSC treatment significantly reduced DSS-induced macrophage infiltration in the colon (Figure 6(a)) but have little influence on B cells in the blood of DSS-induced colitis mice (Figure 6(b)). Simultaneously, DSS induced an elevation of Th17, Th1, and Th2 cells in the spleen of DSS-induced mice, which were moderately reduced by HUC-MSCs (Figures 6(c)–6(e)). On the other hand, HUC-MSCs dramatically increased the number of Tregs in the spleen of DSS-induced mice (Figure 6(f)). These results suggest that HUC-MSCs repressed macrophages, Th1, and Th17 cells, the major active cells during acute inflammation, but increased Treg cells. Among all the immune cells, it appeared that HUC-MSCs remained to have the strongest response on macrophages.

As HUC-MSCs inhibited B16 cells to form tumors in nude mice, which are T cell deficient but retain complete macrophages (see Figures 3(a) and 3(b)), we determined to study how HUC-MSCs inhibited macrophages. To this end, we isolated bone marrow cells from mice and induced the cells into different macrophage populations. The results showed that 49.1% of bone marrow cells were induced into M1 macrophages under the conditional medium; however, the M1 macrophage percentage decreased to 16.7% in the presence of HUC-MSCs (Figure 6(g)). On the other hand, we observed that M2 macrophages were increased by HUC-MSCs from 22.3% to 35.2% (Figure 6(h)). All these results suggest that HUC-MSCs rebalanced the macrophages by increasing M2 but repressing M1. Consistently, we observed that TNF-*α*, a cytokine secreted by M1 macrophages, was decreased, while IL-10, a cytokine secreted by M2 macrophages, was increased by HUC-MSCs (Figures 6(i) and 6(j)). In summary, we reasoned that macrophages are the main effect cells in response to HUC-MSC treatment in colitis-related colon cancer. The inhibition role of HUC-MSCs on tumorigenesis could be attributed to the balance of macrophages, as well as other T cells.

## 4. Discussion

MSC-based therapy is a promising therapeutic approach in the management of several diseases involving tissue inflammation and immune disorders. However, several issues remain unresolved concerning their safety, in particular of the tumorigenesis [24]. It was reported that human umbilical cord-derived mesenchymal stromal cells were safe and feasible for clinical use [25] and able to inhibit breast cancer progression by inducing tumor cell death and suppressing angiogenesis [26]. In this report, we studied the mesenchymal stem cell characteristics and found that HUC-MSCs significantly inhibited tumorigenesis in both B16 cell and colitis-induced colon cancer models. Our results provided strong evidence that MSCs might function on inhibition of tumorigenesis. This added our confidence on the application of MSC-based therapy not only in the inflammation diseases but also in cancers, in particular inflammation-related cancers.

The inhibitory effect of HUC-MSCs on B16 cell-derived tumor in the nude mice astonished us since nude mice lacked T and B cells. However, nude mice retain macrophages which also play an important role on tumorigenesis. Therefore, we determined to decipher whether regulation of macrophages could be effective to inhibiting tumorigenesis. To this end, we employed an AOM/DSS-induced colon cancer model and observed that HUC-MSCs dramatically inhibited the occurrence of colon cancer. Based on these results, we proposed that HUC-MSCs inhibited the tumorigenesis via regulation of macrophages. Indeed, we observed that the macrophage populations and its secreted cytokines were dramatically repressed by HUC-MSCs in both *in vitro* and *in vivo* models. Although we also observed that HUC-MSCs regulated T cells including Th1, 2, 17, and Treg, we observed that the inhibition effect was not as strong as that for macrophages. It is possible that T cell inhibition directly affects tumorigenesis; however, we speculate that HUC-MSCs mainly function on macrophages. The effect of T cell population by HUC-MSCs might also possibly be due to the regulation of macrophages. Further studies are needed to clarify our speculation, although the inhibition of B16 cell-directed tumor model in nude mice provided a clue.

It remains unclear how macrophages regulate tumorigenesis. In this study, we provided evidence that HUC-MSCs inhibited the activation of macrophages. This was demonstrated by the effect of macrophage polarity by HUC-MSCs. It appeared that HUC-MSCs are able to educate macrophages to differentiate into M2 from M1. Recently, M2 macrophages were regarded as an inhibitor for tumors [27–29]. However, other studies reported that the tumor-infiltrated macrophages, after being educated into M2 form, have a potent ability to facilitate tumor growth. These controversial roles of M2 macrophages might imply that M2 macrophages have different subpopulations. Indeed, recent studies have revealed different M2 macrophages including M2a, 2b, 2c, and 2d. Nevertheless, we observed that IL-10, secreted by M2 macrophages, was significantly increased after HUC-MSC treatment. Exosomes, from human bone marrow-derived MSCs, reduce murine colonic inflammation via polarizing M2b macrophages and regulating IL-10 secretion [30]. Therefore, we deserve to study HUC-MSCs on these different subpopulations of M2 macrophages, especially M2b macrophages.

In conclusion, we found that HUC-MSCs alleviate experimental murine colon inflammation and tumorigenesis by decreasing the production of inflammatory cytokines and rebalancing immune cell populations, especially macrophages. Therefore, HUC-MSC therapy could be used as a safe and effective treatment for patients with colitis-associated colon cancer in the future.

## Data Availability

The data that support the findings of this study are available within the article or from the corresponding author upon reasonable request.

## Supplementary Materials

Supplementary 1 Fig. S1: the influence of HUC-MSCs on the main organ indexes of mice. (A–F) The index of the colon, heart, liver, spleen, lung, and kidney in mice injected with HUC-MSCs. HUMSCs were injected once a week.

Supplementary 2 Fig. S2: the homing ability of HUC-MSCs. (A, B) CFSE-labeled HUC-MSCs were intravenously injected to DSS-induced mice, and their presence in the spleen and colon were determined by flow cytometry.
