# Supplementary material for: Umbilical Cord Mesenchymal Stem Cells Ameliorate Inflammation-Related Tumorigenesis via Modulating Macrophages
Source: Stem Cells Int. 2022 Jun 1;2022:1617229. doi: 10.1155/2022/1617229 (PMC9178412; doi:10.1155/2022/1617229)
Supplement: Supplementary 1 — Fig. S1: the influence of HUC-MSCs on the main organ indexes of mice. (A–F) The index of the colon, heart, liver, spleen, lung, and kidney in mice injected with HUC-MSCs. HUMSCs were injected once a week. [file 1617229.f1.pptx]

## Slide 1
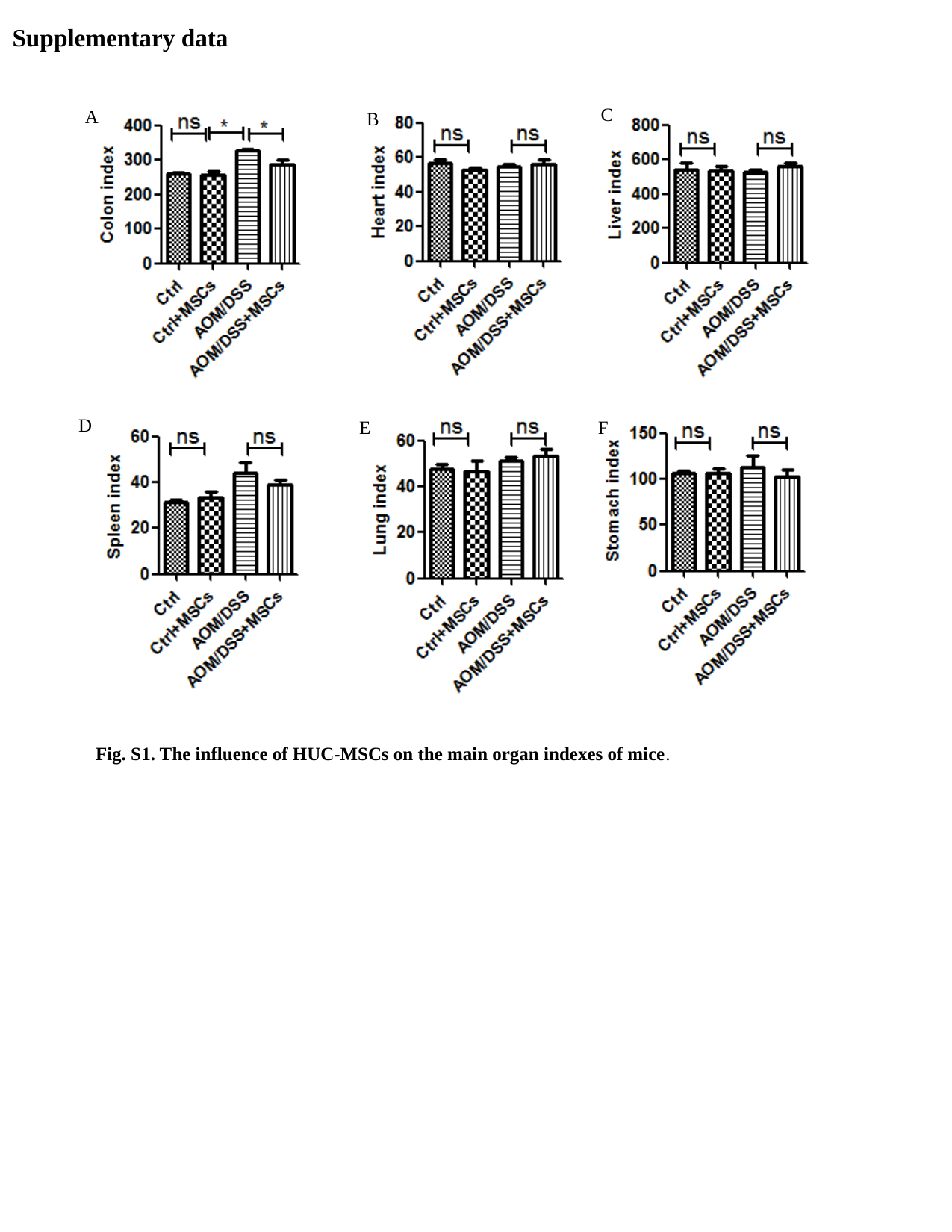

Supplementary data
C
A
B
D
F
E
Fig. S1. The influence of HUC-MSCs on the main organ indexes of mice.
