# Supplementary material for: Umbilical Cord Mesenchymal Stem Cells Ameliorate Inflammation-Related Tumorigenesis via Modulating Macrophages
Source: Stem Cells Int. 2022 Jun 1;2022:1617229. doi: 10.1155/2022/1617229 (PMC9178412; doi:10.1155/2022/1617229)
Supplement: Supplementary 2 — Fig. S2: the homing ability of HUC-MSCs. (A, B) CFSE-labeled HUC-MSCs were intravenously injected to DSS-induced mice, and their presence in the spleen and colon were determined by flow cytometry. [file 1617229.f2.pptx]

## Slide 1
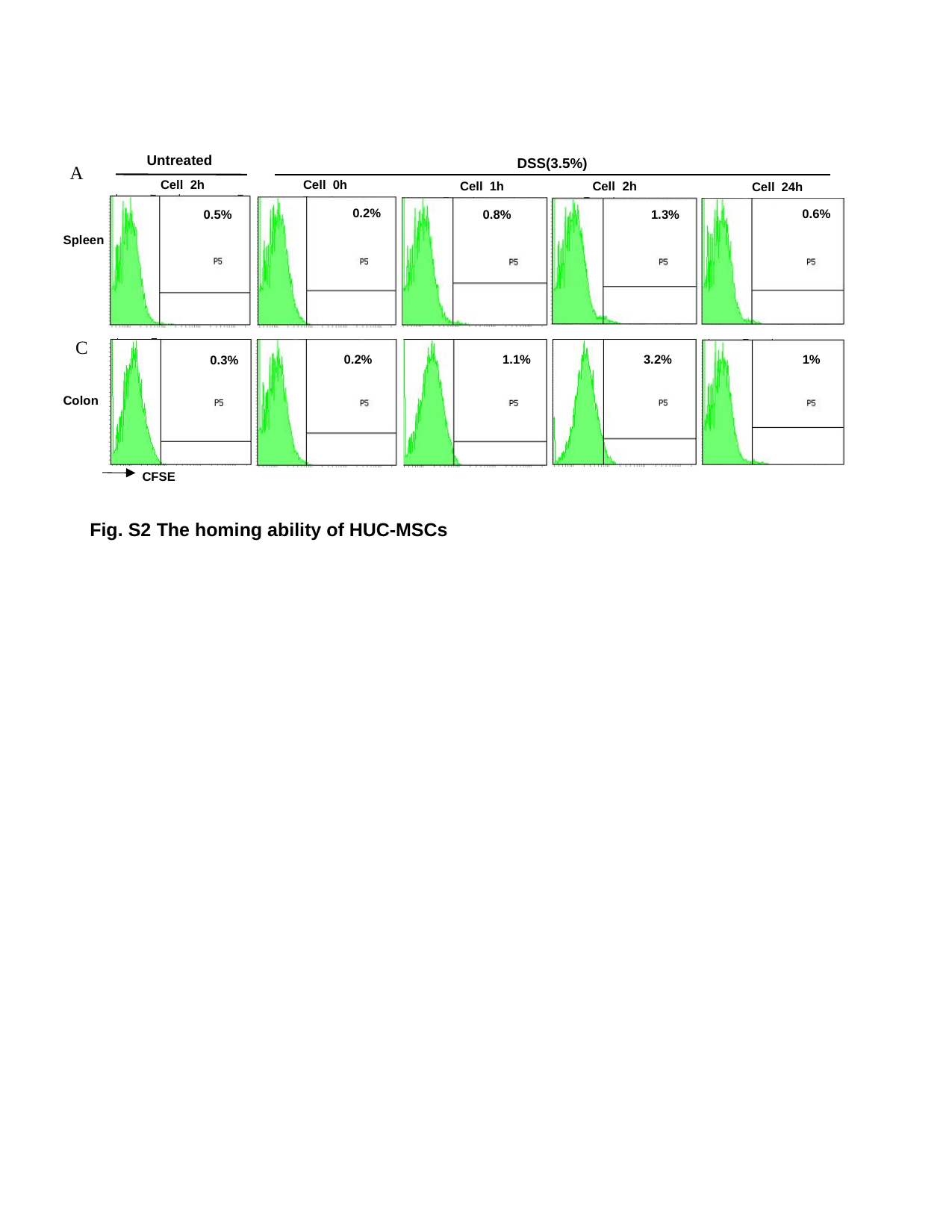

Untreated
DSS(3.5%)
 Cell 2h
A
 Cell 2h
 Cell 0h
Cell 1h
 Cell 24h
 0.6%
 0.8%
 1.3%
 0.5%
0.2%
Spleen
C
 0.3%
1.1%
0.2%
3.2%
Colon
CFSE
1%
Fig. S2 The homing ability of HUC-MSCs
